# BAYAS: simplifying access to Bayesian analysis for biologists

**DOI:** 10.1093/bioinformatics/btaf276

**Published:** 2025-06-13

**Authors:** Christoph Waterkamp, Daniel Hoffmann

**Affiliations:** Bioinformatics and Computational Biophysics, Faculty of Biology, University of Duisburg-Essen, Universitätsstraße 2, Essen, NRW, 45117, Germany; Bioinformatics and Computational Biophysics, Faculty of Biology, University of Duisburg-Essen, Universitätsstraße 2, Essen, NRW, 45117, Germany; Center for Medical Biotechnology, University of Duisburg-Essen, Universitätsstraße 2, Essen, NRW, 45117, Germany; Center for Computational Sciences and Simulation, University of Duisburg-Essen, Universitätsstraße 2, Essen, NRW, 45117, Germany

## Abstract

**Motivation:**

In biological research, complex and noisy biological systems with small effects are often studied with small sample sizes. Such a setting is ideal for Bayesian analysis as it supplements new data with prior knowledge and emphasizes uncertainty quantification. Unfortunately, the proper application of Bayesian analysis requires a degree of computational expertise beyond the training of many biologists.

**Results:**

We have developed BAYAS (BAYesian Analysis Simplified), a web-based tool that provides programming-free access to Bayesian workflows for numerous use cases. BAYAS comes with three modules: *Planning* for Bayesian determination of sample sizes; *Evaluation* for Bayesian analysis of experimental data; *Report* to make analyses transparent and reproducible.

**Availability and implementation:**

BAYAS can be accessed freely at https://bayas.zmb.uni-due.de/app/bayas (server) and https://github.com/GitCJW/bayas_bioinformatics or https://doi.org/10.5281/zenodo.15052467 (source).

## 1 Introduction

For many relevant application scenarios in biological research, the conventional way of doing statistics is null-hypothesis significance testing with *P*-values (NHSTP) (Fisher 1925, Neyman and Pearson 1933). Unfortunately, NHSTP is prone to misinterpretation and can lead to apparent replication failures (Wasserstein and Lazar 2016, Amrhein *et al.* 2019), especially in settings typical for biology with small (costly) samples, small effect sizes, and noisy data (Ioannidis 2005). Under these conditions of scarce and noisy data, cheap alternatives for quantitative estimation such as resampling methods will probably not give more reproducible results than NHSTP. In research with lab animals or clinical samples, these problems can have severe ethical implications, e.g. if animals are sacrificed without gain of knowledge.

Bayesian analysis is an alternative that is better suited to the settings typical for biology, more easily interpretable, and therefore becoming increasingly popular (Kruschke and Liddell 2018). From the perspective of an experimental biologist, the main obstacles to using Bayesian analysis are, first, understanding the main concepts of the Bayesian workflow, and second, acquiring the programming skills needed to implement a Bayesian workflow for the tasks at hand. To overcome these obstacles, we have developed the free software BAYAS (BAYesian Analysis Simplified). BAYAS is designed to facilitate access to Bayesian analyses, in its current version with a focus on Bayesian Generalized Linear Models (GLMs) (McCullagh and Nelder 1989, McElreath 2020). GLMs extend ordinary linear regression: a linear core is transformed to the scale of the expected outcome with an (inverse) link function, and noise is added to complete the likelihood. By using different link and noise functions, GLMs can be flexibly adapted to many application scenarios. BAYAS currently supports binomial, beta, exponential, gamma, (log-) normal, inverse Gaussian, negative binomial and Poisson noise functions, and suitable link functions.

## 2 Design

**Figure 1.**
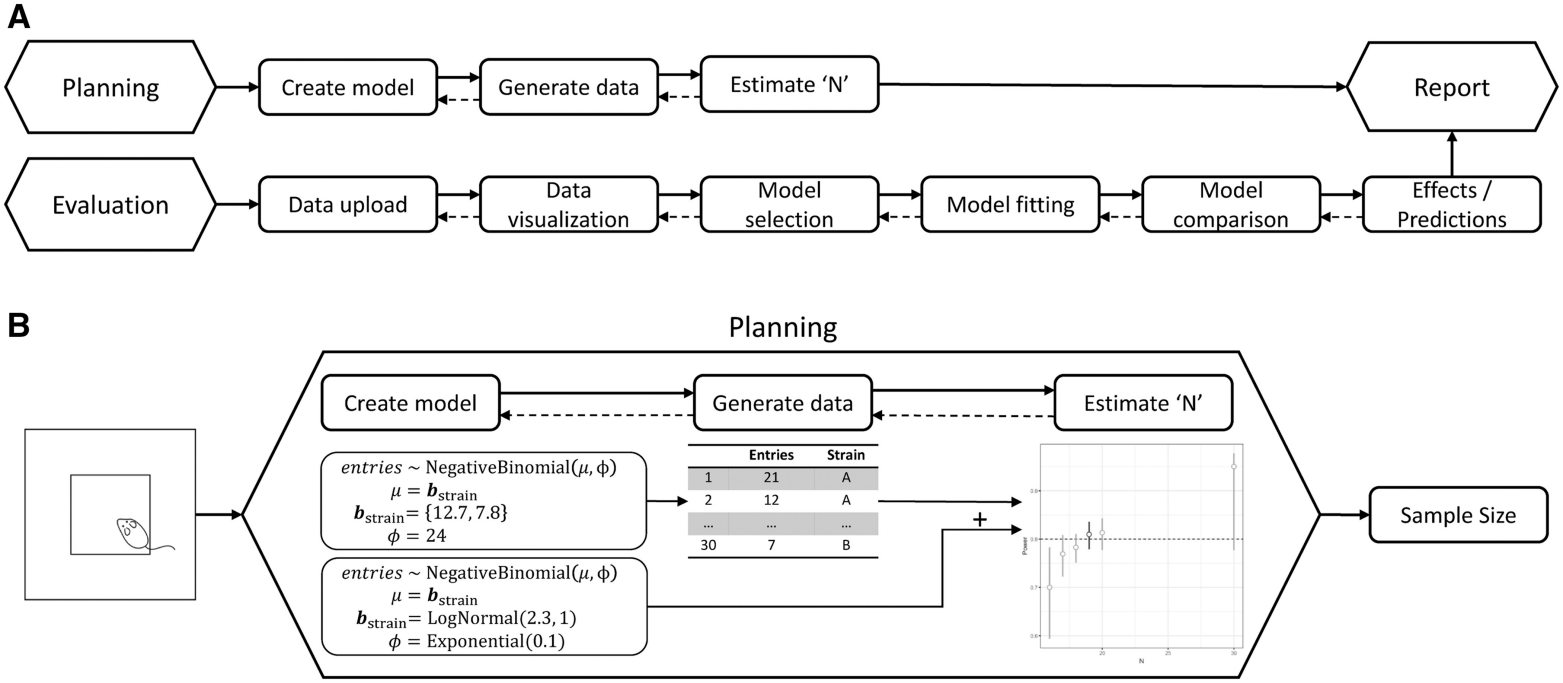
(A) Schematic overview of BAYAS with its three modules Planning, Evaluation, and Report showing the conceptual workflow of the application. (B) Estimation of sample size for an Open Field Test as an example application. In the planning module, two related models are created for data generation (top) and inference (bottom), respectively.

BAYAS is a freely accessible, web-based tool with three modules: *Planning* for model design and sample size planning, *Evaluation* for Bayesian inference, and *Report* for transparent reporting of analyses. These three modules are outlined in the following (Fig. 1).

The *Planning* module is intended to support the design of a statistical model that reflects the properties of the experimental data, and to determine the sample size required for an informative experiment. These two aims are interrelated: only an accurate statistical model will predict a sample size that is suitable for quantitative estimation and testing of system properties. Clearly, for research with animals it is important to find such an accurate model and hence the smallest reasonable sample size. In contrast to the prevailing statistical black-box practice in biology, numerical Bayesian inference is well-suited in this respect as it can be easily applied to a wide range of models, many of which are not readily available for conventional statistics (an example is discussed below).

In the *Planning* module, the user is guided through the definition of two models (see also Supplementary Information): a data generation model and an inference model. The data generation model should capture plausible properties of the data generation process. This model can be used to simulate and to visualize realistic data for a range of trial sample sizes. The simulated data are input to the inference model; the inference model is exactly the Bayesian model that we would later use for analyzing actual data in the *Evaluation* module. For each of the trial sample sizes, multiple samples are simulated and the inference model produces posterior probability distributions, which are tested for achievement of a user-defined *goal* with a user-defined probability (corresponding to the statistical power). A goal could, for instance, be a minimum width (precision) of the posterior, or the exclusion of effect sizes that are practically equivalent to zero. BAYAS identifies the minimum sample size for which the goal is achieved with the chosen power. The whole process can be reproducibly summarized in the Report module.

The *Evaluation* module is the core of BAYAS, the analysis of data by Bayesian statistics. First, the data to be analyzed is uploaded in long format; transformation of wide to long format is supported by BAYAS. Based on the data, BAYAS makes a guess about the type of variable (discrete, continuous, theoretical bounds, etc.). These guesses are transparent and can be corrected by the user. The user then selects one of the variables as response.

Next the user configures an appropriate statistical model, typically a generalized linear model (GLM), including noise term, link function, and linear core. Some of the model components are constrained by the nature of the data (e.g. count versus continuous responses). This insight is used by BAYAS to provide a list of plausible model types. Such a type then has to be refined to a complete model, specified as a mathematical formula. BAYAS aids in this specification by an interactive graphical form. Priors may be proposed automatically or configured by the user.

The posterior distribution, i.e. the probability distribution of the model parameters given the data, is numerically sampled by Hamiltonian Markov Chain Monte Carlo as implemented in Stan (Carpenter *et al.* 2017), and mediated by packages rstanarm (Goodrich *et al.* 2024) or brms (Bürkner 2017).

The outcomes of various checks are reported to make sure that the numerical sampling has been technically valid and that model predictions are consistent with the observed data (posterior predictive checks). If these checks are passed, the user may proceed to evaluate the posterior, e.g. to quantify effect sizes and their uncertainties.

Sometimes several alternative models are conceivable. In that case it is helpful to compare models with respect to their ability to generalize to unseen data. To this end, the *Evaluation* module uses a fast approximation of the leave-one-out cross-validation (Vehtari *et al.* 2022).

To facilitate interpretation of model fits, BAYAS computes effects and makes predictions. Effects are computed between all pairs of categorical predictor values, e.g. the effect of a treatment (treated versus untreated) on animal body mass. All such effects are presented in a matrix. With the fitted model, the user can generate predictions for a given set of predictor values, and inspect such predictions for different input sets.

Effects and predictions are not point estimates but distributions. To summarize these distributions, the user can choose between highest density intervals (HDI) or equal-tailed intervals (ETI). In addition, effects are reported in a suitable form as determined by the model, such as differences, multipliers, log-odds ratios, etc.

The third module, *Report*, is fed by elements selected by the user from the results of the *Planning* or *Evaluation* modules. These elements are collected in a preliminary report, which can be modified and extended at any time. A minimum set of report elements for a reproducible and transparent analysis is prepared by default.

### 2.1 Example

In the following example, we estimate a sample size for the Open Field Test (OFT) (Hall and Ballachey 1932), a widely used behavioral experiment. In the OFT a rodent (here: a mouse) is placed in a square arena, which contains a smaller inner square, the *center* (Fig. 1B). Bolder mice will enter this center more frequently while more anxious mice will tend to avoid the center. The center entries per animal and observation interval are counted. We would like to quantitatively compare two genetic strains of mice, *A* and *B*, with respect to their center entry counts.

Biologists with a conventional training in statistics would normally use one of two tests to identify an effect of mouse strain on center entries in OFT, the *t*-test (Student 1908, Welch 1947), or the Mann–Whitney U test (Mann and Whitney 1947). However, the distributions of center entries violate various assumptions of both tests, especially if we are using the tests to identify the influence of the strain on the central tendency of the distribution. Implying wrong distributional properties can in turn lead to under- or overestimation of sample sizes needed to detect the effect of the genetic strain on center entries. In fact, we have recently shown that assuming a normal model (as in a *t*-test) for OFT center entry counts leads to systematic overestimation of sample sizes in comparison to a more appropriate negative binomial model (Waterkamp *et al.* 2023). In BAYAS it is easy to estimate sample sizes for such a model with the following steps:

- Specify properties of the biological response, for instance that center entries in the OFT are positive count values.
- Set predictor variables, here: mouse strain.
- Define a data generation model to simulate realistic OFT data, and an inference model to infer the strain effect on center entry counts.
- Define a goal that should be achieved with sample size *N*, e.g. a power of 80% for the detection of the strain effect.
- Run data generation and inference models for a range of *N* values, using the data generation model as ground truth that should be inferred correctly.
- BAYAS homes in on minimum *N* that satisfies the defined goal.

In the end BAYAS recommends a sample size N=19 for the OFT experiment under the given assumptions. With the conventional approach of a two-sided power *t*-test (significance level of 5%, power of 80%, effect size of 4.86 and pooled standard deviation of 4.49), we would have obtained a recommended sample size of N=29.

We have used the open field data (72 data points in total) mentioned in Waterkamp *et al.* (2023) to obtain the empirical mean and variance (and the corresponding dispersion parameter ϕ for the negative binomial noise distribution) as inputs for both sample size determination in BAYAS and the power *t*-test.

A walkthrough video that details the technical steps for this example is integrated in BAYAS and can be accessed easily on the starting page of the software.

## 3 Technical implementation

BAYAS is implemented in R, and relies on several key R packages, notably the *shiny* package (Chang *et al.* 2024) for the building of interactive websites, and packages for Bayesian analysis such as *rstan* (Stan Development Team 2024), *rstanarm* (Goodrich *et al.* 2024), and *brms* (Bürkner 2017).

## 4 Discussion and outlook

BAYAS makes it easier for biologists to analyze their data with Bayesian methods by removing one layer of difficulty, namely programming. However, there is still a second layer, the conceptual understanding of Bayesian models and workflows. In BAYAS we therefore provide explanatory material, such as walkthrough videos or short texts with visuals, and we are in the process of compiling a library of educational texts and videos on concepts and biological use cases.

So far, BAYAS focuses on Bayesian GLMs, a flexible and versatile class of models that according to our experience covers many use cases biologists have in practice. Multilevel or hierarchical models will be a worthwhile addition as the partial pooling of information between groups of samples and adaptive regularization often lead to superior performance (Kruschke 2014, McElreath 2020). Unfortunately, multilevel models are also more difficult to formulate, to check and to interpret.

Bayesian modeling lends itself to the causal network analysis (McElreath 2020), hence another attractive extension that could build on the existing BAYAS infrastructure would be a module for such analyses or their integration into the Planning (Qin 2024) and Evaluation modules.

Real-world data are often incomplete, requiring specific processing for effective modeling. Currently, BAYAS automatically restricts the dataset to complete cases and notifies the user. Future updates will include alternative methods for handling missing values, such as treating them as unknown parameters.

BAYAS guides users through complex workflows. With the *Report* module such workflows can be made transparent and reproducible. In the future, we will offer standardized reports that fully comply with guidelines such as ARRIVE (Percie du Sert *et al.* 2020), BARG (Kruschke 2021), or similar.

One of the advantages of Bayesian inference over conventional statistics is the use of prior information, making inferences more efficient. Ideally, one would choose a sequential experiment design (DeGroot 2005, McMichael *et al.* 2021) in which data are collected step-by-step, with the posterior being updated in each step and then used as prior for the next step. Such a sequence would be stopped once sufficient knowledge has been acquired. This optimally efficient sequential design would e.g. save numerous lives of lab animals. Unfortunately, this design will in general also take more time and is also more difficult to manage in practice. A realistic option that would make good use of Bayesian updating is the inclusion of historical data drawn from the relevant literature in the design of priors (Shen *et al.* 2023).

Finally, the quick pace of developments in artificial intelligence opens further options for significant enhancements of BAYAS. For instance, it is already possible to have a large language model generate code (e.g. in Stan) for a Bayesian model from a textual description of a problem. To our experience, such automated code generation is not yet reliable but this may change in the near future and then could become a valuable extension of the model-building process in BAYAS.

A further application of artificial intelligence techniques that could be integrated in BAYAS relatively easily is amortized inference (Gershman and Goodman 2014). This would speed up the most time-consuming tasks such as sample size determination.

## Supplementary Material

btaf276_Supplementary_Data
