# Supplementary material for: BAYAS: simplifying access to Bayesian analysis for biologists
Source: Bioinformatics. 2025 Jun 13;41(6):btaf276. doi: 10.1093/bioinformatics/btaf276 (PMC12199779; doi:10.1093/bioinformatics/btaf276)
Supplement: btaf276_Supplementary_Data [file btaf276_supplementary_data.pdf]

BAYAS Statistics Report

March 21, 2025

1 Planning of experiment "OFT_entries"

This chapter describes a statistical model that represents a real-world phenomenon (e.g. a biological experiment). Reported *seeds* bring quasi-random number generation into defined initial state for reproducibility. The model is also used to determine the sample size for a given set of requirements.

1.1 Formula

The formula is a mathematical representation of the model and contains important information about relations between variables. A formula of the structure "$Y \sim X$" means that a variable Y has a distribution X. Often such formulas are used to describe our prior knowledge (or *prior* for short). E.g. if you read $\alpha \sim \text{Normal}(0, 1)$ this means that *we know* that a variable α follows a normal distribution with mean 0 and standard deviation 1. Variables with underlined names are vectors of one or more elements.

There are two versions of the model used for estimating the sample size N, which differ in the priors. The *data generation* model has highly informative priors that reflect a realistic size of the effect of interest, and the model is used to generate samples. These samples are fed into the *inference* model, which is used to infer probability distributions of model parameters. These distributions are key to the sample size determination, e.g. if the probability distribution of a target parameter is sufficiently narrow, our sample size is large enough. The inference model should also later be the one that will be applied to the actual data.

1.1.1 Data generation model

$$\begin{aligned} entries &\sim \text{Negative\_Binomial}(\mu, \phi) \\ \mu &= \underline{b_{strain}} \\ \underline{b_{strain}} &= \{12.7, 7.8\} \\ \phi &= 24 \end{aligned}$$

$\underline{strain} = \{\text{a}, \text{b}\}$

1.1.2 Inference model

$$
\begin{aligned}
entries &\sim \text{Negative\_Binomial}(\mu, \phi) \\
\mu &= \underline{b_{strain}} \\
\underline{b_{strain}} &\sim \{\text{Log-normal}(2.3, 1), \text{Log-normal}(2.3, 1)\} \\
\phi &\sim \text{Exponential}(0.1)
\end{aligned}
$$

$\underline{strain} = \{\mathrm{a}, \mathrm{b}\}$

1.2 Definition of (other) variables

Variables describe a particular set of data and are either *categorical* or *numerical.* A categorical variable can take one of a limited, fixed number of possible values, representing different categories or groups. These values are typically qualitative and non-numeric, although they can be encoded as numbers for analysis purposes. Each categorical variable is defined by its *elements* and their frequencies of occurrence in a single batch. When a variable is *capped*, batches are cut to a desired length. For example: The variable *sex* with an *explicit* occurrence of the elements *female*(1) and *male*(2) means that *female* occurs once and *male* twice. If the dataset length is 5 and the variable is *capped*, the variable will have elements (*female*, *male*, *male*, *female*, *male*). In this capped example the set (*female*(1), *male*(2)) or, explicitly, (*female*, *male*, *male*) is repeated twice, and the second repeat is reduced by one *male* to fit into the desired length of 5 elements.

To allow for random variation in the occurrence of elements (in this case, the frequency is set to *equal* by default), a randomization parameter is used to specify the percentage of variation between elements. For example, if *male* and *female* are equally distributed, but with a variation of 10%, the difference between the occurrences of the two elements can be at most 10%, e.g. 10 times *male* and 11 times *female.*

1.2.1 'strain'

Type: *Categorical*
Elements: *a, b*
Frequency of elements: *Equal*
Capped: *Yes*
Randomize frequency: *No*

1.3 Parameter seeds

1.4 Example data

A sample dataset showing the variables used and their values. The values of the response variables (the leftmost one) are generated using the generative model.

ϕ	Generate data	Inference
	2981	3249

Table 1: Used seeds for parameter 'ϕ'.

$\underline{b_{strain}}$	Generate data	Inference
a	-8947	-3124
b	5983	790

Table 2: Used seeds for parameter '$\underline{b_{strain}}$'.

Response seed: *123*

entries	strain
20	a
6	b

Table 3: Minimum example dataset of '*OFT_entries*'.

1.5 Sample size determination

Sample size determination is the process of calculating the number of observations or participants needed in a study to reliably detect or exclude an effect. Unlike conventional approaches such as the power t-test, we here use a simulation-based approach with two parameters. The *power* is the probability of achieving the formulated goals, and the *approximate number simulations* controls the number of simulations for a certain sample size. *Goals* are user-defined conditions that must be met for the sample size to be considered sufficient. In general, such a condition involves an effect between parameters of the model, i.e. a difference between parameters, think e.g. of the difference between average body masses of male and female mice as the effect of sex on the parameter average body mass μ. This effect is defined by the sum of the *first group* of parameters minus the sum of the *second group*. Remember that parameters, and thus effects, are not given by single values, but by distributions. Considering, for example, 95% of the *probability mass* of these distributions can lead to a more robust approach.

ROPE (Region Of Practical Equivalence) refers to a range of values that are interpreted as de facto equal (e.g. because all values in ROPE are biologically indistinguishable or fall below the limit of detection of a measurement instrument). For example, you could consider body masses between two groups of mice as practically equivalent if their difference in grammes lies in interval [-1,+1] (which thus would be the ROPE). All

values within this range will be treated as *equal*. A possible condition for the estimation of the sample size is to have a sample size large enough to see a meaningful effect, i.e. an effect of a size that with high credibility *excludes* the ROPE. Alternatively, if it is more probable that there is no meaningful effect, a useful condition for the sample size is the *inclusion* of the probability density within the ROPE. *Lower* and *upper* define the ROPE limits.

As probability distributions become more narrow – uncertainty decreases – with increasing sample size, another useful condition for the sample size could be that the *precision* (i.e. the width of a probability density distribution) should be better than a given value.
Power: *0.8*
Max N: *30*
Approximate number simulations: *400*
Seed: *123*

1.5.1 Sample size determination goals

Goal *non_zero*, see figure 1
Parameters in group 'A': $b_{strain:a}$
Parameters in group 'B': $b_{strain:b}$
Goal type: *ROPE*
ROPE type: *Exclude*
ROPE lower bound: *0*
ROPE upper bound: *0*
Probability mass: *0.95*%

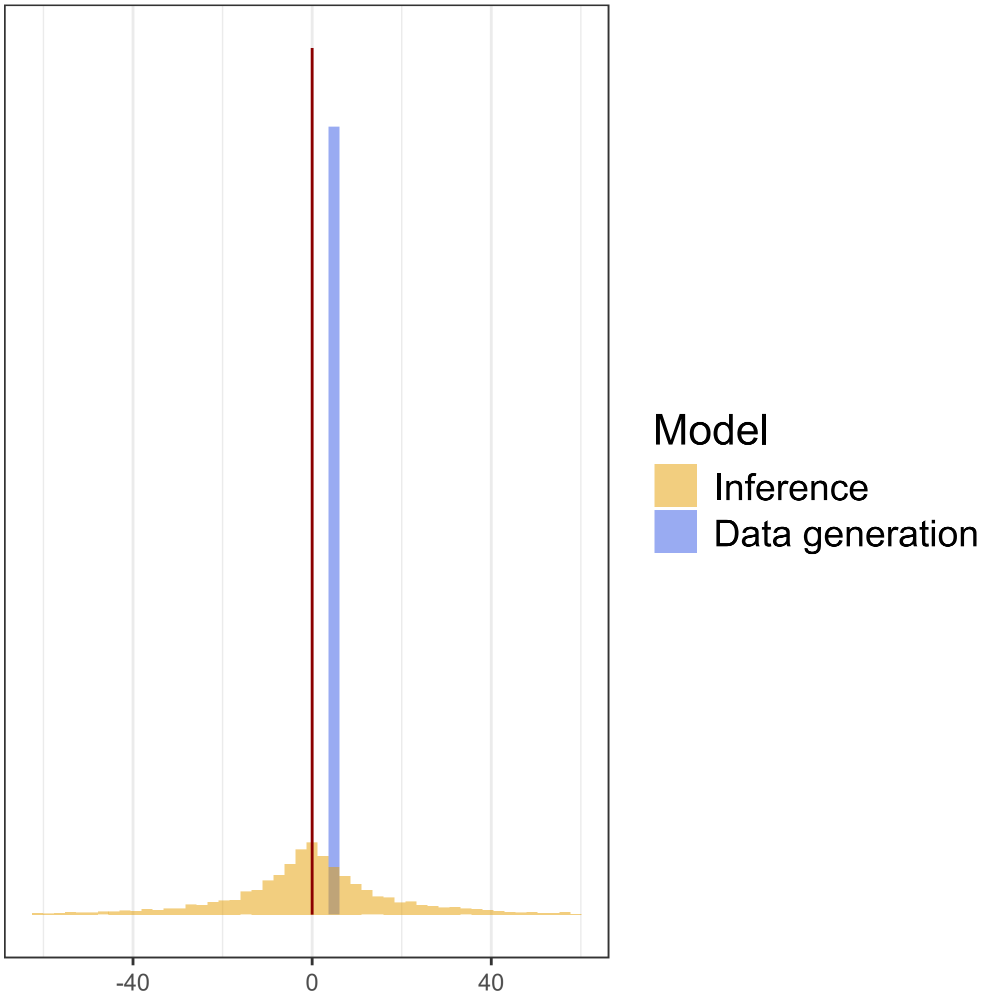

Figure 1: A single goal ‘non_zero’ of the sample size determination. Histograms of the expected difference of the parameters of the *inference* model and the *data generation* model before sampling. The red rectangle represents the ROPE.

1.5.2 Sample size determination result

If a sample size can be determined that fulfills the conditions, this will reported. Because this is a simulation-based approach where the number of simulations is indirectly controlled by the user, the uncertainty of the estimated power for a given sample size is quantified by a 90% credible interval. The reported sample size is the total number of observations aggregated over all groups.Result of sample size determination of ‘OFT_entries’, see figure 2 and table 4
Potential N: *19*
90%-credible interval of power: *0.779 - 0.836*

Power interval width: *0.0566*
Number of simulations for this N: *520*

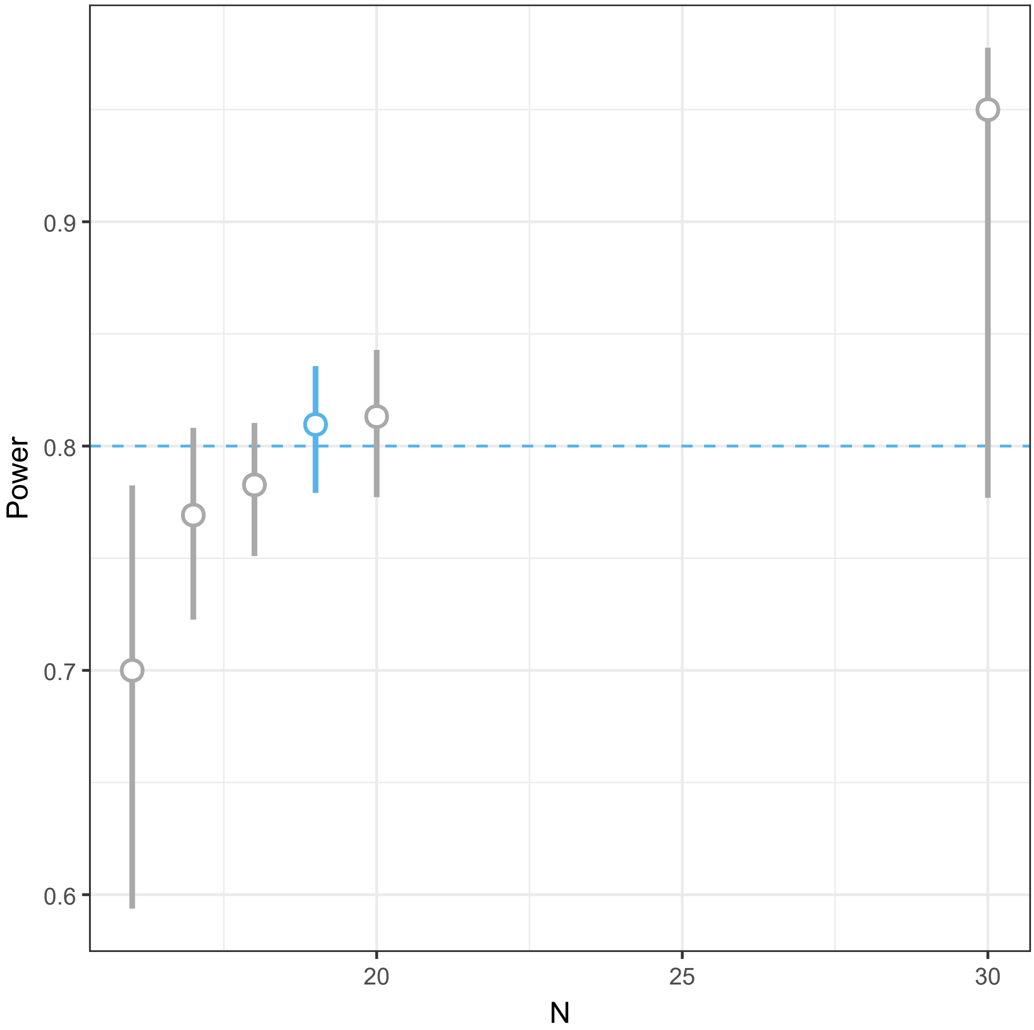

Figure 2: Sample sizes (horizontal axis) simulated in sample size determination ('OFT_entries'), and power (vertical axis) reached for these sample sizes. Each circle marks the fraction of trials with the indicated sample size in which the goal was achieved. Vertical error bars are 90% credible intervals of power. The blue dashed line shows the desired power and the blue circle/bar shows the smallest of the tested sample sizes at which this power is probably surpassed. See table 4 for more details on the number of simulations per sample size.

N	#Simulations	Power	Certain
30	20	0.95	FALSE
16	60	0.7	FALSE
17	260	0.769	FALSE
20	380	0.813	TRUE
19	520	0.81	TRUE
18	520	0.783	TRUE

Table 4: Sample size determination of experiment 'OFT_entries'. Simulated N with its number of simulations and empirical power.
